# Interleukin-6, Age, and Corpus Callosum Integrity

**DOI:** 10.1371/journal.pone.0106521

**Published:** 2014-09-04

**Authors:** Brianne M. Bettcher, Christa L. Watson, Christine M. Walsh, Iryna V. Lobach, John Neuhaus, Joshua W. Miller, Ralph Green, Nihar Patel, Shubir Dutt, Edgar Busovaca, Howard J. Rosen, Kristine Yaffe, Bruce L. Miller, Joel H. Kramer

**Affiliations:** 1 University of California San Francisco, Neurology Department, Memory and Aging Center, San Francisco, California, United States of America; 2 Rutgers University, Department of Nutritional Sciences, New Brunswick, New Jersey, United States of America; 3 University of California Davis Medical Center, Department of Pathology and Laboratory Medicine, Sacramento, California, United States of America; 4 University of California San Francisco, Department of Psychiatry, Neurology, and Epidemiology and Biostatistics, San Francisco, California, United States of America; University of Cambridge, United Kingdom

## Abstract

The contribution of inflammation to deleterious aging outcomes is increasingly recognized; however, little is known about the complex relationship between interleukin-6 (IL-6) and brain structure, or how this association might change with increasing age. We examined the association between IL-6, white matter integrity, and cognition in 151 community dwelling older adults, and tested whether age moderated these associations. Blood levels of IL-6 and vascular risk (e.g., homocysteine), as well as health history information, were collected. Processing speed assessments were administered to assess cognitive functioning, and we employed tract-based spatial statistics to examine whole brain white matter and regions of interest. Given the association between inflammation, vascular risk, and corpus callosum (CC) integrity, fractional anisotropy (FA) of the genu, body, and splenium represented our primary dependent variables. Whole brain analysis revealed an inverse association between IL-6 and CC fractional anisotropy. Subsequent ROI linear regression and ridge regression analyses indicated that the magnitude of this effect increased with age; thus, older individuals with higher IL-6 levels displayed lower white matter integrity. Finally, higher IL-6 levels were related to worse processing speed; this association was moderated by age, and was not fully accounted for by CC volume. This study highlights that at older ages, the association between higher IL-6 levels and lower white matter integrity is more pronounced; furthermore, it underscores the important, albeit burgeoning role of inflammatory processes in cognitive aging trajectories.

## Introduction

Aging is associated with myriad cognitive and structural brain changes that were once assumed to be immutable processes; however, current conceptualizations underscore the contribution of modifiable risk factors [1], [2], with increasing emphasis on the role of the immune system in aging outcomes [3], [4]. While numerous associations have been found between inflammation and neurodegenerative disease [5], less is known about how inflammatory mediators might relate to the cognitive neuroscience of aging.

Although the pro-inflammatory cascade involves numerous mediators, interleukin-6 (IL-6) represents one of the more widely researched inflammatory factors. IL-6 is released by peripheral and central nervous system cells, including adipose tissue, glia, and neurons, and is expressed at low levels in healthy adults. Circulating blood levels of IL-6 increase with age [6] and may have negative effects if elevated for a sustained period. Specifically, IL-6 is strongly associated with a spectrum of vascular-mediated diseases (e.g. cardiovascular disease, atherosclerosis) [7] that increase vulnerability to cerebrovascular events [8] and may hasten cognitive decline in older adults [9]. An important consideration, however, is how IL-6 levels might relate to specific brain tissue and whether the strength of this association increases with age. Inflammation more broadly induces changes in vascular permeability [10] and myelin morphology [11], both of which may contribute to alterations in white matter integrity. In accord with these proposed mechanisms, recent studies have demonstrated that higher levels of inflammation are related to reduced fractional anisotropy (FA) in white matter tracts of healthy elders [11], particularly in the corpus callosum [12], [13]. No studies to date, however, have examined factors that might account for the relation between IL-6 levels and white matter integrity, nor have prior investigations clarified how this association might change with increasing age. This is particularly important given the significant remodeling of the immune system in older adults [14], as the link between IL-6 levels and brain structure may become more prominent over time.

The current study addresses pressing gaps in the literature by examining the relation between IL-6 levels and white matter integrity, and assessing whether it is a) moderated by age and b) independent of traditional vascular risk factors and blood markers. Considering the association between corpus callosum (CC) integrity and aging [15], vascular risk factors [16], and inflammation [12], [13], [17], we chose the CC as our primary region of interest. However, in order to assess the possibility of more extensive white matter alterations, we also examined whole brain associations with IL-6. We hypothesized that mean levels of CC integrity would be lower among subjects with higher IL-6 levels than among subjects with lower IL-6, and magnitudes of this association would increase with age. We also assessed whether higher IL-6 levels were related to a cognitive index strongly linked with white matter integrity in older adults (i.e. information processing speed) [18], thereby solidifying a connection between IL-6 and both structure and behavior. We predicted that higher levels of IL-6 would be associated with slower processing speed.

## Methods

### Participants (Table 1)

**Figure 1:**
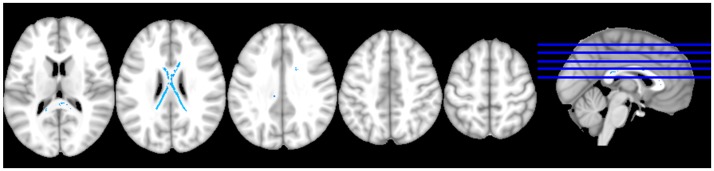
Whole brain analysis of IL-6 levels and white matter integrity. Whole brain TBSS analysis displayed at p≤01, using TFCE and corrected for multiple comparisons. The figure shows white matter regions inversely associated with IL-6 levels, thereby displaying areas in which higher IL-6 levels were associated with lower fractional anisotropy. Significant results were primarily restricted to the body and splenium, and extend into the genu of the corpus callosum.

**Figure 2:**
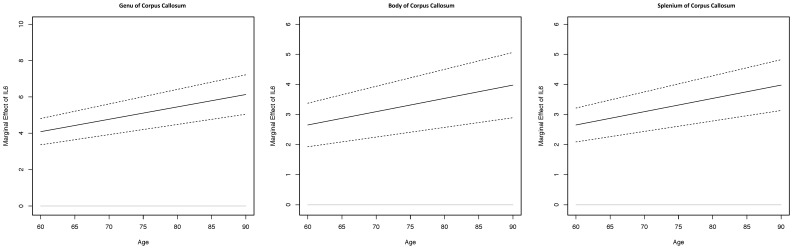
Displays slopes for the association between IL-6 and white matter integrity in the genu, body, and splenium of the corpus callosum across age (solid line) accompanied by 95% confidence intervals (dashed lines). IL-6 marginal effects are adjusted for demographic variables and vascular factors/blood markers.

**Figure 3:**
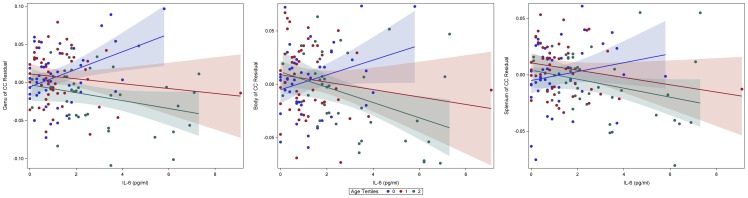
Scatterplots display the association between IL-6 levels and white matter integrity, as a function of age tertiles (for descriptive purposes). White matter fractional anisotropy variables (i.e. genu, body, splenium of the corpus callosum) were regressed over covariates to create a residualized variable. Age was divided into tertiles; ‘0’ reflects the lowest ages in our sample (<67 years), ‘1’ reflects the middle third of ages (67–71.9 years), and ‘2’ indicates the highest ages (≥72 years).

A sample of 152 healthy, community dwelling older adult participants was selected from the University of California, San Francisco Memory and Aging Center database based on the availability of plasma blood markers of interleukin-6 (IL-6) as well as diffusion tensor imaging. Both evaluations occurred within a 90-day period. Participants were recruited from our larger NIH Aging and Cognition study (ages 62–90), and were reviewed in a screening visit, which entailed an informant interview, neurological examination, and cognitive testing. Inclusion as a “healthy” participant was based on several criteria, including a Mini-Mental State Exam score of ≥26, Clinical Dementia Rating score of 0, and no subject or informant report of cognitive decline during the previous year. Participants were excluded if they had a major psychiatric disorder, neurological condition affecting cognition (e.g. large vessel infarct), dementia or mild cognitive impairment, substance abuse, systemic medical illnesses (e.g. cancer), current medications likely to affect CNS functions, or current depression (Geriatric Depression Scale Score >15/30) [19]. Notably, one individual’s data was removed (see Laboratory Measures section below) due to concerns of underlying illness (n = 151). The study was approved by the UCSF committee on human research, and all subjects provided written, IRB-approved informed consent before participating. In compliance with data sharing plans, both summary data (Table 1; Figure 1; Figure 2) and raw data (Figure 3 scatterplot) are provided in the manuscript.

**Table 1:** Participant Characteristics, Laboratory Markers, and Neuroimaging Indices.

	N	Mean (SD)	Range
**Demographics/Clinical Characteristics:**			
Age (Years)	151	71.6 (5.7)	62–87
Gender (% Female)	151	57.0	
Education (Years)	151	17.5 (2.1)	11–22
GDS (Total Score)	142	2.7 (2.9)	0–15
Homocysteine (umol/L)	150	8.3 (3.5)	2.4–30.5
IL-6 (pg/ml)	151	1.8 (1.7)	0.0–9.1
Body Mass Index	136	25.4 (3.7)	18.0–37.2
History of Hypertension (%)	143	39.4	
History of Smoking (%)	144	51.0	
History of Hypercholesterolemia (%)	144	50.7	
Diabetes (%)	144	4.2	
**Structural and Diffusion Imaging:**			
Corpus Callosum (Total Volume)	144	2867.39 (403.44)	1831.00–3989.00
Corpus Callosum (Mean FA)	151	0.63 (0.03)	0.53–0.70
*Splenium*		0.71 (0.03)	0.63–0.77
*Body*		0.61 (0.04)	0.51–0.69
*Genu*		0.57 (0.04)	0.45–0.65
**Cognitive Evaluation:**			
MMSE (Total Score)	151	29.4 (0.9)	26.0–30.0
Processing Speed (Composite Score)	150	1.5 (1.4)	−0.2–8.7

Abbreviations: GDS = Geriatric Depression Scale; IL-6 = interleukin-6; FA = fractional anisotropy.

### Measures

#### Body Mass and Vascular Risk

Body mass index (BMI) was calculated as follows: [weight (kg)/height (m)^2^], and analyzed as a continuous variable. All available information regarding medical and health history was garnered via participant self-report. Health variables included lifetime history (yes/no) of the following: smoking, hypertension, hypercholesterolemia, and diabetes.

#### Laboratory Measures

All participants were queried regarding recent illnesses in the past week (e.g. influenza, respiratory infection, etc). One individual’s data was subsequently removed from all analyses due to concerns that they may have had an acute underlying illness. Fasting blood was collected into serum separator tubes between 8∶00 am and 10∶00 a.m. for all participants, and left to clot at room temperature for 30–60 min, and into EDTA plasma tubes. The blood was then centrifuged at 2500 rpm (1300–1800) at room temperature for 15 min. Plasma and serum were stored at −80°C until analysis. IL-6 was measured using a Quantikine ELISA kit from R&D systems (Minneapolis, MN). Homocysteine, an independent risk factor for vascular disease [20], was measured by HPLC with post-column fluorescence detection [21]. IL-6 and homocysteine were measured in the research laboratory of Dr. Ralph Green at the University of California Davis Medical Center (Sacramento, CA).

#### Neuroimaging

MRI scans were obtained within 3 months of the participants’ blood draw. Scans were conducted on a 3.0 Tesla Siemens (Siemens, Iselin, NJ) TIM Trio scanner equipped with a 12-channel head coil located at the UCSF Neuroscience Imaging Center. Whole brain images were acquired using volumetric magnetization prepared rapid gradient-echo sequence (MPRAGE; TR/TE/TI = 2300/2.98/900 ms, α = 9°). The field of view was 240×256 mm, with 1×1 mm in-plane resolution and 1 mm slice thickness. Diffusion imaging data were acquired via a spin-echo, echo planar imaging sequence with 55 slices 2 mm thick (TR/TE = 8000/109 ms, FOV = 220 mm, matrix = 100×100) in two series. One series contained diffusion gradients and 64 diffusion directions (b = 0 and b = 2000 s/mm^2^, 1 average) while the other had no diffusion gradients and 6 diffusion directions (b = 0, 10 averages).

FreeSurfer: The T1 MPRAGE structural MR images were analyzed using the FreeSurfer image analysis suite, which is documented and freely available for download online at: http://surfer.nmr.mgh.harvard.edu. FreeSurfer is a surface-based structural MRI analysis tool that segments white matter and tessellates both gray and white matter surfaces [22]. Previous publications have provided detailed descriptions and validation of the software [23], [24]. After initial automated segmentation through FreeSurfer version 5.1, each subject’s image was individually checked for quality and accuracy of segmentation. Inaccuracies in grey and white matter segmentation, as well as inaccurate pial boundaries and residual skull fragments were manually corrected with FreeSurfer’s built-in editing software. These cases were then reprocessed to take edits into account and final volumes were recalculated. Intracranial volume (ICV) was calculated based on FreeSurfer’s own eTIV (estimated total intracranial volume) metric, which uses atlas normalization as well as the relationship between the linear transform to MNI305 space and ICV.

Tract-Based Spatial Statistics: DTI data were eddy current corrected using FMRIB’s Diffusion Toolbox (FSL 4.1.6; http://fsl.fmrib.ox.ac.uk/fsl) [25]. Brain extraction and binary brain mask creation took place using the b0 image through the FSL Brain Extraction Tool. Fractional Anisotropy (FA) maps were created based on the diffusion tensor modeling results from FSL DTIFIT. Whole brain white matter analysis was conducted using FSL’s TBSS protocol [26]. All subjects’ FA data were registered using the nonlinear registration tool FNIRT [27] to the IXI Aging DTI Template [28] masked by a study-specific binary averaged image. The mean FA and mean FA skeleton were created from the study sample. Each subject’s aligned FA data was then projected onto the mean FA skeleton and the resulting data fed into voxelwise cross-subject statistics.

#### Cognitive Measures

Cognitive testing was obtained within 3 months of the participants’ blood draw and neuroimaging scan. Considering the strong association between both aging and slower processing speed [29], as well as lower fractional anisotropy and slower processing speed [18], [30], participants were administered a series of processing speed tasks, and were asked to make rapid judgments about stimuli presented on the laptop display. The tasks have been validated in previous studies [31]. Briefly, the tasks included the following: animal matching, word rhyming, word judgment, and word pronunciation tasks. Animal matching requires assessment of whether the word presented on a screen is an animal or not; word rhyming requires evaluation of whether two four-letter words presented on a screen rhyme; word judgment necessitates assessment of whether the 3-letter stimuli presented are real words versus non-words; finally, the word pronunciation task requires the subject to choose which stimuli sounds like a real word (e.g. “doon” vs “filt”; correct answer is “doon” as it sounds similar to the real word, “dune”). For data reduction purposes, a scaled response latency z-score was calculated for each task based on the mean and standard deviation time among a sample of young adult controls (n = 40; 17 males age 23.6±2.5 years). We created a single mean z-score based on all tasks, with higher factor scores reflecting slower information processing speed.

### Statistical Analyses

#### Covariate Selection

Using correlation analyses, demographic and blood marker variables were identified as covariates for statistical models if they were significantly associated with either the predictor variable (IL-6) or primary region of interest (ROI) (CC; *p*<.05). Age was mean centered at 71.6 years.

#### Whole Brain

We hypothesized an inverse association between IL-6 and corpus callosum FA; however, given the strikingly limited literature on this topic, we initially conducted a whole brain analysis to ensure that additional regions strongly associated with IL-6 levels were not excluded. Whole brain statistical analyses were carried out using FSL’s Randomise, and covariates included demographic variables. Results for this analysis were considered significant if they survived Threshold-Free Cluster Enhancement (TFCE) corrected at p≤0.01 [32]. We chose to employ the conservative p-value to minimize the number of subsequent comparisons conducted, and further focus on areas that displayed the most robust effects.

#### ROI

We employed the JHU ICBM-DTI-81 white matter labels [33] to mask areas of the white matter skeleton, corresponding to major white regions. We examined the genu, body, and splenium of the CC as primary ROI’s. It is unclear whether IL-6 levels associates with specific regions of the CC or the entire interhemispheric tract; however, considering the differential vulnerability of the CC to underlying neurodegenerative disease processes [34], [35] and metabolic factors [16], we chose to examine these sections individually. Notably, we elected to include the entire ROI for each section of the CC rather than extracting significant voxels from the whole brain analysis in order to employ a more conservative analytic approach.

Mean FA values for each white matter region were calculated from the white matter skeleton.

In addition, the combined volumes of the following FreeSurfer regions were considered in exploratory analyses: Corpus Callosum (CC) Posterior, CC Mid-Posterior, CC Central, CC Mid Anterior, and CC Anterior.

#### Regression Analyses

We fit general linear models with age and IL-6 as predictors, demographics and vascular risk factors/blood markers as covariates, and an interaction term between age and IL-6 as the primary variable of interest. CC ROI’s (separately) served as the dependent variables. To quantify the marginal effect of IL-6 in the presence of an interaction between age and IL-6, we estimated this marginal effect of IL-6 as a function of both the main effect and interaction terms on an equally spaced grid of age values. Confidence intervals were based on standard error estimates obtained using the delta method. This allowed us to address whether the magnitudes of association between IL-6 and white matter outcomes differed as a function of participant’s age. Given that several of the independent variables are known to be associated with each other [12], the corresponding regression coefficients may be poorly determined and exhibit high variance [36]. Ridge regression analyses are thought to provide protection against potential inflation of variance in the estimates, thus alleviating effects of multicollinearity [36]. We therefore conducted a follow-up ridge regression analysis in order to assess the robustness of our conclusions.

In order to evaluate whether IL-6 levels were related to processing speed, we conducted an additional analysis in which IL-6 served as a primary predictor (including both the main effect and the interaction term), and the processing speed score served as the dependent variable.

#### Exploratory Analyses

Two exploratory analyses were conducted. Given the strong association between processing speed and white matter [18], we investigated whether CC FA accounted for the association between IL-6 levels and processing speed using a linear regression analysis.

For the second exploratory analysis, we evaluated whether IL-6 was related to corpus callosum volume, in addition to FA values, using a linear regression analysis.

All linear analyses were conducted using SAS (version 9.4). Ridge regression was conducted based on a ridge regression function implemented in the R project for statistical computing (http://www.r-project.org).

## Results

### Covariate Selection

Lower FA of the CC was associated with: higher age, male gender, history of hypertension, higher homocysteine levels, higher BMI, and a history of ever smoking. Higher IL-6 levels were associated with older age, history of hypertension, higher BMI, and higher homocysteine levels. Education, diabetes (n = 4) and past history of hypercholesterolemia were not related to IL-6 levels or CC integrity. Thus, only age and gender (demographics); history of hypertension and smoking, and BMI (vascular risk factors); and homocysteine (vascular marker) were included in models.

### Whole Brain Analysis (Figure 1)

After controlling for demographic variables, higher IL-6 levels were associated with significantly lower FA of the CC, predominantly in the splenium and body regions (*p* = .01). Although isolated, scattered voxels of the right middle corona radiata were significantly associated with IL-6, no consistent pattern was noted bilaterally, nor was the association observed in anterior/posterior regions. No other regions were significantly (*p*≤.01) positively or inversely associated with IL-6.

### ROI Regression Analyses (Figure 2, Figure 3)

Linear regression analyses indicated that the interaction between age and IL-6 was significant for the splenium [F(1,126) = 7.23; t = −2.69; unstandardized beta = −.0006; 95% CI = −.0011 to −.0002; *p* = .008], body [F(1,126) = 9.56; t = −3.09; unstandardized beta = −.0009; 95% CI = −.0015 to −.0003 *p* = .002], and genu [F(1,126) = 8.62; t = −2.94; unstandardized beta = −.0009; 95% CI = −.0016 to −.0003; *p* = .004] of the corpus callosum, after controlling for all covariates. Main effects for IL-6 and age were not significant. In terms of confound vascular markers, male gender [F(1,126) = 4.49, *p* = .04] and higher BMI were [F(1,126) = 5.78, p = .02] associated with lower FA in the splenium, hypertension [F(1,126) = 3.66, *p* = .058] with lower FA in the body, and BMI for lower FA in the genu [F(1,126) = 7.92; *p* = .006).

We followed-up the previous analysis with a ridge regression analysis; this permitted us to determine the robustness of our results, given the noted association between our vascular markers and IL-6. Ridge analyses evidenced comparable findings, with the interaction between age and IL-6 significant for all three sections of the CC (spenium: *p = *.002; body and genu: *p* = .001).

The aforementioned analyses suggest that a synergistic effect of older age and higher IL-6 predicts *lower* white matter integrity in the splenium, body, and genu of the CC. In order to further describe the interaction between IL-6 (continuous variable) and age (continuous variable), we plotted the magnitude of IL-6 effects relative to selected age bins (i.e. 60–90 years), by 5 year increments for the genu, body, and splenium of the CC. As shown in Figure 2, the magnitude of IL-6 effects on the genu, body, and splenium increases with age, thus demonstrating a synergistic effect of age and IL-6 on white matter integrity.

In addition, to garner a better understanding of the directionality of the relationship between age, IL-6 and white matter integrity, we also plotted the association between IL-6 and white matter integrity as a function of age tertiles (Lowest Tertile ‘0’<67.0 years; Middle Tertile ‘1’ = 67.0–71.9 years; Highest Tertile ‘2’≥72 years) for descriptive purposes. We employed tertiles in order to maintain equal size groups while also viewing IL-6 effects across the age span; notably, similar results were found when dividing age into decades (e.g. 60’s, 70’s, 80’s). As shown in Figure 3, individuals in higher age groups tended to display a negative slope, with higher IL-6 levels yielding lower white matter integrity. Notably, individuals at lower age ranges (i.e. lowest tertile) evidence a different pattern, in which higher IL-6 levels were related to relatively better integrity across all three white matter regions.

### Cognitive Analyses

An initial examination of the main effect of Il-6 levels on processing speed indicated that higher levels of IL-6 were associated with slower information processing speed [F(1, 146) = 3.99; t = 2.00; unstandardized beta = 0.1414; 95% CI = 0.0016 to 0.2813; *p* = .0475). After controlling for demographic variables and vascular factors/blood markers, the interaction between IL-6 levels and age was associated with processing speed [F(1,125) = 4.90; t = 2.21; unstandardized beta = .0255, SE = .01; 95% CI = .0027 to.0484; *p* = .03], suggesting that at higher ages and higher IL-6 levels, processing speed is slower. Main effects for age and IL-6 were not significant for the final model (including all covariates), nor were other confound covariates.

### Exploratory Analyses

We assessed whether CC FA accounted for the association between the IL-6-age interaction and speed. After controlling for all covariates and CC FA, the overall interaction between IL-6 and age was no longer a significant predictor of speed [F (1, 124) = 3.29, *p* = .07], although it remained a trend.

Finally, we also examined the association between IL-6 levels and CC volume. All regions of the CC were related to IL-6 in the aforementioned analyses, thus we focused on total volume. After controlling for all covariates and intracranial volume, IL-6 levels were not significantly related to total CC volume [F (1, 119) = .67, *p* = .66].

## Discussion

The current study suggests that while vascular markers may influence the pernicious correlation between IL-6 levels and corpus callosum integrity, older age robustly and independently modulates this association. Specifically, the interaction between age and IL-6 remains predictive of CC FA after controlling for vascular risk factors/blood indices; these analyses suggest that the reduction of CC integrity associated with IL-6 is larger at older ages relative to that at younger ages. To our knowledge, this is the first study to examine possible moderators of the relationship between IL-6 levels and white matter integrity in older adults. Results from this study add to a growing body of literature suggesting that ‘typical’ aging is quite heterogeneous, and propelled in part by a combination of metabolic [37] and inflammatory factors [38] that may be amenable to future therapies.

Our findings further suggest multi-factorial associations between IL-6 levels, brain structure, and cognition that are modified by the aging milieu. Importantly, the influential role of age in our study highlights that a linear process may not adequately capture the deleterious relationship between IL-6 levels and white matter integrity. Older age has been extensively correlated with higher levels of circulating inflammatory markers as well as a host of changes in both innate and adaptive immune functioning [39], [40]; while immunocompetence should not be considered a static process, our study suggests that negative associations with white matter may be conferred at higher levels of IL-6, and the magnitude of this effect strengthens with older age. Thus, older age combined with higher levels of IL-6 appears to yield greater risk for lower white matter integrity. Interestingly, when plotting the association between IL-6 and white matter integrity using tertiles of age, IL-6 levels appeared to be positively related to white matter at younger ages. Although we employed these scatterplots to further visualize the interaction between our two continuous variables, the age at which a deleterious association between IL-6 and white matter integrity manifests remains unclear; moreover, the possibility of a different and even beneficial association between IL-6 levels and brain health at younger ages should be further explored. In order to fully explicate the association between IL-6 and white matter integrity across the older adult age span, further research is needed.

Consistent with our predictions, IL-6 was primarily associated with CC FA in the whole brain analysis, suggesting that this interhemispheric tract may be not only vulnerable to aging and vascular processes, but also inflammation-associated alterations. In addition, our appraisal of white matter volume yielded no significant associations with IL-6 levels, indicating that these findings are not driven solely by CC atrophy. This is consistent with prior literature highlighting the role of diffusion metrics as an early marker of white matter changes rather than solely a downstream effect of volume loss [15], [41]. Catalysts underlying the aging process (e.g. cellular senescence) may play a pivotal role in altering how and when risk factors extend negative effects [42]; however, longitudinal studies are critical to understanding these potentially influential factors.

An additional goal of the study was to clarify whether vascular factors solely accounted for the association between IL-6 and CC FA. Higher BMI and a history of hypertension remained predictive of CC FA in our study, but did not fully account for the IL-6-white matter relationship. Notably, a pro-inflammatory process by its very nature utilizes vascular mechanisms to reach target cells and tissues; however, this does not necessarily imply that its association with white matter integrity is due to vascular damage. In line with this thinking, study analyses underscore that pro-inflammatory processes relate to white matter integrity, independent of traditional vascular conduits. Our results thereby imply a more complicated picture, in which both vascular markers and inflammatory markers may contribute to ongoing white matter changes in typical aging. Non-vascular mechanisms by which inflammation may directly relate to white matter integrity or specific brain tissue remain unclear and cannot be determined from the current observational study. Importantly, however, our findings suggest that examining the association between inflammation and brain structure in isolation may yield an incomplete and inaccurate model that does not fully capture the influential role of age in the process.

An important behavioral analog to the IL-6 narrative is that individuals with higher IL-6 levels displayed markedly slower information processing speed than those with lower IL-6. Similar to the DTI findings, this association increased as a function of age. Although processing speed was evaluated due to its strong link with interhemispheric and fronto-parietal white matter [43], the parallel relationship between IL-6 and both white matter and cognition suggests a possibly more extensive and compelling role for inflammation in cognitive aging.

The current study displays numerous strengths, including the use of multimodal imaging and multiple indices of vascular risk. In addition, we also employed ridge regression techniques to assess the robustness of our interaction effects. Ridge regression alleviates the negative effects of correlation among predictors on properties of estimates; in our study, comparable findings using both methods suggest that our results are quite robust. However, it is prudent to highlight limitations. First, we employed a peripheral inflammatory marker, which may not reflect the CNS environment. Although basic science research suggests a link between peripheral markers and CNS functioning [44], direct comparisons to CNS levels cannot be made based on the current data. In addition, we only employed one diffusion metric in our white matter analyses; although we chose the most commonly used measure of white matter integrity (fractional anisotropy), future studies should examine associations with other metrics, including radial diffusivity and axial diffusivity. Finally, our study was cross-sectional in nature and thus cannot address issues of causality. Longitudinal studies are needed to provide a more definitive understanding of not only the progression of inflammatory markers over time in healthy older adults, but also the temporal associations between inflammation and structural brain changes. While we have clearly pitched a dynamic association between age, IL-6 levels, and white matter integrity, the directionality of these relationships cannot be gleaned from observational data. Thus, it is possible that the role of IL-6 levels is ancillary to another primary process not measured in the current study.

In conclusion, study findings demonstrate that the detrimental association between IL-6 levels and corpus callosum white matter integrity is moderated by age, and this relationship is independent of traditional vascular risk factors and blood markers. This study highlights the important, burgeoning role of immunological processes in understanding cognitive aging trajectories.
